# Diagnostic Performance of Pancreatic Cytology with the Papanicolaou Society of Cytopathology System: A Systematic Review, before Shifting into the Upcoming WHO International System

**DOI:** 10.3390/ijms23031650

**Published:** 2022-01-31

**Authors:** Ilias P. Nikas, Tanja Proctor, Svenja Seide, Stylianos S. Chatziioannou, Jordan P. Reynolds, Dimitrios Ntourakis

**Affiliations:** 1 School of Medicine, European University Cyprus, Nicosia 2404, Cyprus; sergecha93@gmail.com (S.S.C.); d.ntourakis@euc.ac.cy (D.N.); 2 Institute of Medical Biometry, University of Heidelberg, 69120 Heidelberg, Germany; proctor@imbi.uni-heidelberg.de (T.P.); seide@imbi.uni-heidelberg.de (S.S.); 3 Department of Laboratory Medicine and Pathology, Mayo Clinic, Jacksonville, FL 32256, USA; reynolds.jordan@mayo.edu

**Keywords:** pancreas, endoscopic ultrasound-guided fine needle aspiration (EUS-FNA), immunohistochemistry, pathology, molecular, cancer, pancreatic intraductal neoplasms, neuroendocrine tumors, sensitivity and specificity, diagnosis

## Abstract

The Papanicolaou Society of Cytopathology (PSC) reporting system classifies pancreatobiliary samples into six categories (I–VI), providing guidance for personalized management. As the World Health Organization (WHO) has been preparing an updated reporting system for pancreatobiliary cytopathology, this systematic review aimed to evaluate the risk of malignancy (ROM) of each PSC category, also the sensitivity and specificity of pancreatic FNA cytology using the current PSC system. Five databases were investigated with a predefined search algorithm. Inclusion and exclusion criteria were applied to select the eligible studies for subsequent data extraction. A study quality assessment was also performed. Eight studies were included in the qualitative analysis. The ROM of the PSC categories I, II, III, IV, V, VI were in the ranges of 8–50%, 0–40%, 28–100%, 0–31%, 82–100%, and 97–100%, respectively. Notably, the ROM IVB (“neoplastic—benign”) subcategory showed a 0% ROM. Four of the included studies reported separately the ROMs for the IVO subcategory (“neoplastic—other”; its overall ROM ranged from 0 to 34%) with low (LGA) and high-grade atypia (HGA). ROM for LGA ranged from 4.3 to 19%, whereas ROM for HGA from 64 to 95.2%. When the subcategory IVO with HGA was considered as cytologically positive, together with the categories V and VI, there was a higher sensitivity of pancreatic cytology, at minimal expense of the specificity. Evidence suggests the proposed WHO international system changes—shifting the IVB entities into the “benign/negative for malignancy” category and establishing two new categories, the “pancreatic neoplasm, low-risk/grade” and “pancreatic neoplasm, high-risk/grade”—could stratify pancreatic neoplasms more effectively than the current PSC system.

## 1. Introduction

Fine-needle aspiration (FNA) of the pancreas—performed mostly with endoscopic ultrasound (EUS-FNA)—is a safe, minimally-invasive, and specific diagnostic procedure. EUS-FNA can effectively triage the aspirated material for cytomorphologic, biochemical, and molecular pathology evaluation, facilitating the diagnosis of pancreatic solid and cystic lesions and improving patient care [1,2,3]. The Papanicolaou Society of Cytopathology (PSC) reporting system uses a standardized approach, classifying pancreatobiliary samples into the following categories: I, nondiagnostic; II, negative; III, atypical; IV, neoplastic (consisting of two subcategories: IVB, neoplastic—benign; IVO, neoplastic—other); V, suspicious for malignancy; and IV, malignant. While the IVB category comprises mostly serous cystadenoma (SCA), the IVO is rather heterogeneous including intraductal papillary mucinous neoplasms (IPMNs) and mucinous cystic neoplasms (MCNs) of any grade, besides solid neoplasms exhibiting malignant potential, like pancreatic neuroendocrine tumors (PanNETs) and solid pseudopapillary neoplasms (SPNs) [4,5]. The goals of the PSC reporting system implementation are to summarize the morphologic criteria and provide risk stratification of each reporting category, as well as to incorporate radiologic, biochemical, and ancillary technique findings, and facilitate the communication among physicians [4,5].

Of interest, the World Health Organization (WHO) has been preparing an updated system for reporting pancreatobiliary cytopathology. Proposed changes include eliminating the “neoplastic: benign” PSC subcategory, while shifting SCA and lymphangioma interpretations into the “benign/negative for malignancy” WHO category; replacing the “neoplastic—other” PSC category with two new WHO categories, the “pancreatic neoplasm, low-risk/grade” and “pancreatic neoplasm, high-risk/grade”, encompassing the interpretations of IPMN or MCN with low-to intermediate and high-grade dysplasia, respectively; and moving the PanNETs and SPNs from the “neoplastic—other” PSC subcategory into the WHO category “positive for malignancy”, aligning with the recent WHO Classification of the Digestive System Tumors [6,7]. 

As no systematic review of studies presenting their results with the PSC reporting system has been published in the literature to date, the main outcomes of our study were to evaluate the ROM of each PSC category (I–IV) while reporting pancreatic FNAs, in addition to the sensitivity and specificity of pancreatic FNA cytology with the PSC system. We believe this analysis is important before implementing the upcoming WHO system into everyday practice.

## 2. Materials and Methods

### 2.1. Search Strategy

This systematic review was performed according to the Preferred Reporting Item for Systematic Review and Meta-Analysis (PRISMA) Statement [8]. Five databases (PubMed, Embase, Scopus, Web of Science, Cochrane Library) were investigated up until 31 August, 2020, with the following search algorithm: “Papanicolaou AND (system OR classification OR terminology OR nomenclature OR reporting OR guideline*) AND pancrea*”. The same term was applied once more on the PubMed database to update the search and include any eligible articles until August 2021. No filters were used, while the duplicates were removed with the Paperpile reference manager (https://paperpile.com/app, accessed on 31 August 2020). 

### 2.2. Study Selection

The study inclusion and exclusion criteria are presented in Table 1. Two authors (I.P.N, S.S.C.) first performed the initial title–abstract selections in an independent manner, using the Rayan App (https://www.rayyan.ai/, accessed on 31 August 2020) [9]. They subsequently performed a full-text evaluation of all eligible articles derived from the selection step, arriving at the final list of articles to be used for data extraction. For any discrepancies, the two authors reached a consensus.

### 2.3. Data Extraction

The following data were extracted in an Excel^®^ file: first author, year, country, study design, study period, research setting, lesion types included (solid; cystic; both), type of intervention (e.g., EUS-FNA), needle size, follow-up type (histology; histology and clinical/radiological follow-up), follow-up duration, time of classification with the PSC system (at initial diagnosis or reclassification for the study), total number of patients, total number of FNA cases, and number of cases with follow-up. In addition, the total number of cases reported under each PSC reporting category, and the number and percentage of them with a positive outcome, were also extracted. 

### 2.4. Study Quality Assessment

The study quality assessment was conducted with the Quality Assessment of Diagnostic Accuracy Studies (QUADAS-2) tool [10,11]. Risk of bias under each domain (patient selection; index test; reference standard; flow and timing) was assessed as low, unclear, or high. 

## 3. Results

### 3.1. Literature Search

**Figure 1:**
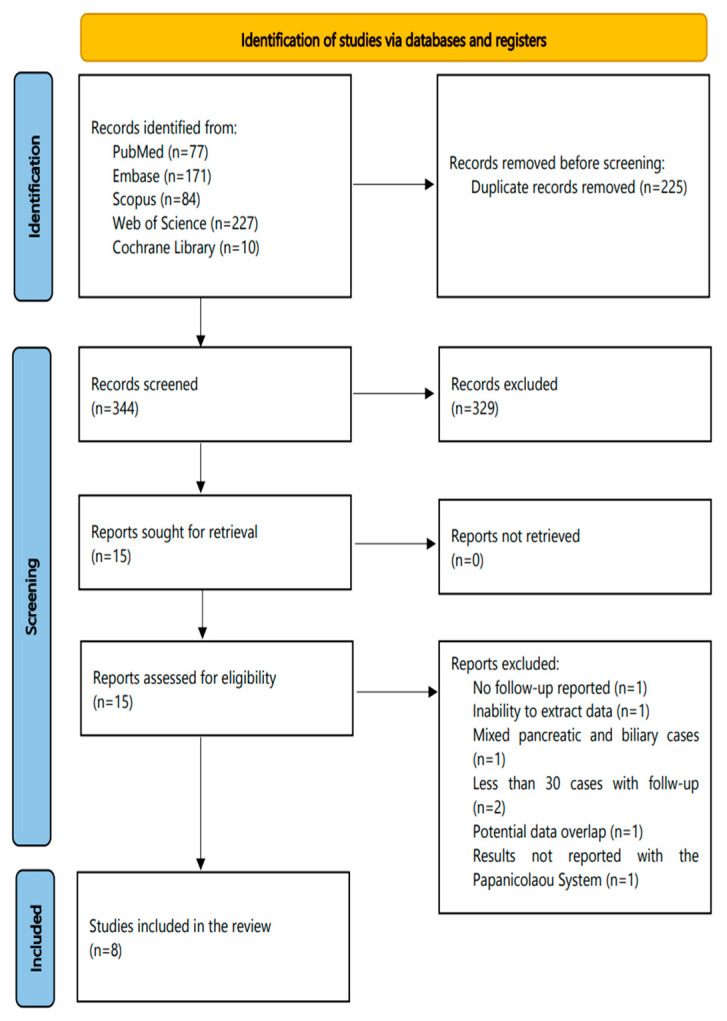
The flowchart of this systematic review.

The flowchart of this systematic review is shown in Figure 1. The initial search (31 August, 2020) identified 563 articles (PubMed, 71; Embase, 171; Scopus, 84; Web of Science, 227; Cochrane Library, 10); of them, 225 were duplicates. The extra PubMed search revealed 6 more eligible studies (PubMed, 77 studies in total) until 31 August, 2021. Subsequently, a total of 344 studies were screened in a title-abstract fashion. Following this step, 15 studies were considered as eligible for full-text evaluation. The latter resulted in the exclusion of seven more studies, resulting in eight eligible studies that were further analyzed in this review. 

### 3.2. Characteristics of the Included Studies

The main characteristics of the eight eligible studies are displayed in Table 2. These were published between 2014 and 2020, while they were most often reported from authors employed in the USA (n = 5). Most studies had a retrospective (n = 6), rather than a prospective design (n = 2). Study period ranged from one year to 15 years and 8 months. All but one were single center studies (n = 7), while all (n = 8) were performed in a university setting. Seven of them stated they only used EUS-FNA for all their included cases. Needle size ranged from 19 to 25 G. Most studies evaluated both solid and cystic lesions (n = 6), whereas two of them only cystic. Six studies considered both histology and clinical/radiological information as follow-up, while two studies only histology. Categorization with the PSC system was performed at the initial diagnosis in four studies, whereas cases were reclassified from the initial reporting in three studies. The total number of patients included was 2254 and the total number of pancreatic FNA cases 2448, whereas follow-up was available for 1959 patients.

### 3.3. Study Quality Assessment

In the study quality assessment (Table 3), no study was regarded as having low-risk in all four domains of the QUADAS-2 tool. In the “patient selection” domain, two studies reported only cystic, rather than a mixture of solid and cystic lesions from a specific period, thus were considered of high bias risk. All studies were rated with an unclear risk of bias in the domain “reference standard”, as pathologists often know the result of the index test (cytology) before interpreting the histology sample (the relevant signaling QUADAS-2 question, under this domain, says: “Were the reference standard results interpreted without knowledge of the results of the index test?” [10,11]). Lastly, in the “flow and timing” domain, six studies were considered having a high-risk of bias, as they had different reference standards across their included cases (either histology or clinical/radiological follow-up).

### 3.4. ROM of the PSC Reporting System

Table 4 shows the number of cases diagnosed under each PSC category, the number of them found positive with the reference standard used in each study (either histology or histology and clinical/radiological), and the percentage of positive cases confirmed with the reference standard/total number of cases (ROM). In the “nondiagnostic” category I, the ROM ranged from 8 to 50%, while in the “negative” category II from 0 to 40%, the “atypical” category III from 28 to 100%, the “neoplastic” category IV from 0 to 31% (also the “neoplastic—other” subcategory IVO from 0 to 34%), the “suspicious for malignancy” category V from 82 to 100%, and the “malignant” category VI from 97 to 100%. Of interest, in the “neoplastic—benign” subcategory IVB, the ROM was 0% in all three studies reporting separately the case numbers from the IVB category [13,14,15].

Notably, a few studies divided the “neoplastic—other” category into neoplasms with low (LGA) and high-grade atypia (HGA). All found the ROM was much higher neoplasms with HGA [12,13,14,18]. Hoda et al. reported the ROM was just 4.3% (2/46 cases) in the LGA, whereas 90% (18/20 cases) in the HGA subcategory [13]. Similarly, Sung et al. found that the ROM was 19% (16/84 cases) in the LGA and 95.2% (20/21 cases) in the HGA subcategory [14]. Lastly, Smith et al. [18] and Gilani et al. [12] reported a ROM of 13% (10/78 cases) and 17% (11/65 cases) in the LGA subcategory, whereas a ROM of 64% (7/11 cases) and 100% (3/3 cases) in the HGA subcategory, respectively.

### 3.5. Sensitivity and Specificity of Pancreatic FNA Cytology Using the PSC Reporting System

Table 5 shows the sensitivity and specificity of pancreatic FNA cytology reported with the PSC system, as displayed in the eligible studies of this systematic review. Different cut-offs were used each time, to decide if cytology would be considered positive or negative for this analysis. When only category VI was regarded as positive, sensitivity ranged from 12.50 to 73.26% and the specificity from 96.55 to 100%. When both categories V and VI were considered positive, sensitivity ranged from 29.17 to 82.89% and the specificity from 85.7 to 100%. Notably, a few studies showed that considering as cytologically-positive the subcategory IVO-with HGA (together with the categories V and VI) resulted in higher sensitivity, at almost no expense of the test specificity [12,13,14,18].

## 4. Discussion

The PSC reporting system was developed with the aim to improve communication among clinicians and offer guidance for personalized management, through providing risk stratification and supporting a multimodal approach that incorporates cytomorphologic, radiologic, biochemical, immunochemical, and molecular findings [4,5]. For instance, CEA cystic fluid levels more than 192 ng/mL and/or the presence of a KRAS mutation support the diagnosis of mucinous neoplastic cyst, while a GNAS mutation the diagnosis of IPMN [5,20,21,22,23]. This standardized system has been reported to reduce nondiagnostic and atypical interpretations [12,24]. However, its implementation has received some criticism, especially for the controversial subcategory “neoplastic—other”, which encompasses lesions of variable malignant potential (IPMNs and MCNs of all grades, also PanNETs and SPNs) [6].

**Figure 2:**
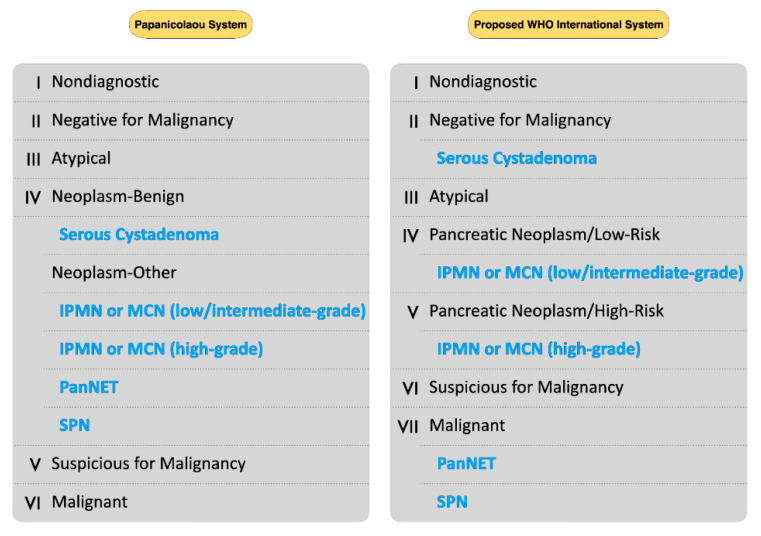
Comparison of the Papanicolaou Society of Cytopathology (PSC) System and the proposed WHO international system for reporting pancreatobiliary cytology. The neoplasms highlighted with blue are shifted into their new categories of the upcoming WHO system.

The upcoming WHO international system aims to align cytology reporting with the recent WHO classification of the digestive system tumors, facilitating the communication among physicians of different specialties [6,7]. In this system, both “neoplastic—benign” and “neoplastic—other” PSC subcategories have been eliminated, whereas two new WHO categories—the “pancreatic neoplasm, low-risk/grade” and “pancreatic neoplasm, high-risk/grade”—have been established, encompassing IPMN or MCN with low-to intermediate and high-grade dysplasia, respectively. In addition, SCA have been shifted from the “neoplastic—benign” PSC subcategory into the “benign/negative for malignancy” WHO category, while PanNETs and SPNs have been moved from the “neoplastic—other” PSC subcategory into the WHO category “positive for malignancy” (Figure 2) [6,7]. As the WHO cytology reporting system has not officially been published yet, evidence concerning its diagnostic value is still lacking. In a recent study, Hoda et al. retrospectively reclassified their previously published data into this upcoming WHO system, aiming to calculate the ROM of each WHO category. They reported that the ROM was 7.7% for the WHO category I, 1% for the category II, 28% for the category III, 4.8% for the category IV, 60% for the category V, and 100% for both WHO categories VI and VII [6]. However, this has been the only study published so far concerning this system.

The evidence presented in our systematic review, although limited, supports the proposed changes in the upcoming WHO international system. All included studies reporting “neoplastic—benign” results showed that the latter had a 0% ROM (Table 3). The most common interpretation under this category is the SCA, a benign neoplasm most often followed-up rather than operated [4,25]. Thus, incorporation of these interpretations into the “benign/negative for malignancy” new WHO category seems reasonable. Furthermore, four of the included studies reported separately the ROMs for “neoplastic—other” with LGA and HGA [12,13,14,18]. ROM for LGA ranged from 4.3 to 19%, whereas ROM for HGA ranged from 64 to 95.2%. Notably, when the subcategory “neoplastic—other” with HGA was regarded as cytologically-positive (together with the categories “suspicious” and “malignant”), this resulted in higher sensitivity of the pancreatic FNA, at almost no expense of the specificity (Table 5) [12,13,14,18]. Hence, evidence suggests the proposed WHO reporting system, with its two new “pancreatic neoplasm, low-risk/grade” and “pancreatic neoplasm, high-risk/grade” categories, could potentially stratify pancreatic neoplasms (conservative management vs potential surgery) more effectively than the existing PSC system, as high-risk/grade cystic lesions have been associated with a much higher ROM. The criteria to detect HGA in pancreatic cystic fluid cytology—high nuclear/cytoplasmic ratio, nuclear membrane irregularities, hyper- or hypochromasia, and necrosis [26,27]—have been reported to be sensitive and specific to predict HGD or malignancy in histology [28], while demonstrating good interobserver reproducibility [29,30]. Notably, a recent immunohistochemical marker, the Das-1, has shown to be highly accurate detecting high-risk mucinous pancreatic cysts, especially when combined with cytology [31,32]. Our study found the ROM of the “atypical” category ranged from 28 to 100%. Reporting under this category has been linked with significant interobserver variability; while reasons for an “atypical” interpretation include sample degeneration, limited cellularity, prominent reactive or dysplastic changes, gastrointestinal contamination, or inexperience of the pathologist [33]. Rapid on-site evaluation (ROSE) could reduce sampling artifacts during EUS-FNA [34]. However, it seems to be of reduced value in pancreatic cystic lesions [35]. Reduction in “atypical” interpretations could be reached by applying ancillary techniques like immunohistochemistry or next-generation sequencing, asking help from experts, or performing a repeat FNA [33,36,37,38].

As expected, both “suspicious” and “malignant” categories exhibited high ROM (82–100% and 97–100%, respectively) in our study. Ancillary testing performed on the cytologic material may help to additionally reach a specific malignant diagnosis; for instance, BCL-10 immunopositivity supports the diagnosis of acinar cell carcinoma [39], while specific IHC panels may help identify PDAC variants [40,41] or metastases to the pancreas [42,43].

This study has some important limitations. The number of included studies was small, mostly of retrospective design and short duration. There was significant heterogeneity, especially in the patient selection—a mixture of studies with solid and cystic or only cystic lesions—and follow-up types, as some studies used only histology, whereas most a mixture of histology and clinical/radiologic follow-up. While relying on histologic-only follow-up could overrate the ROM, especially in the categories I–III of the PSC reporting system due to partial verification bias, a mixture of histology and clinical/radiologic follow-up could have the opposite effect [13]. Moreover, the eligible studies used different criteria to define their positive histologic outcome; some used only PDAC or other cancers, while others added HGD, PanNETs, or IPMNs of any grade. For these reasons, we decided not to perform a prevalence or diagnostic accuracy meta-analysis to calculate the pooled ROM and sensitivity/specificity, respectively, because its results could be misleading to the scientific community.

## 5. Conclusions

A standardized reporting system helps stratify patients undergoing pancreatic FNA. Whereas heterogeneity was present among the studies included in our systematic review, evidence supports the changes proposed in the upcoming WHO international system. Future studies will examine the ability of the latter to provide high diagnostic accuracy and effective risk stratification of patients with pancreatic lesions.

## Figures and Tables

**Table 1:** Study inclusion and exclusion criteria.

**Inclusion Criteria**
Original studies
Studies reporting pancreatic FNA cases of solid and/or cystic lesions
Studies reporting pancreatic FNA cases with available follow-up
Studies performed on humans
**Exclusion Criteria**
Studies written in a language other than English
Animal model or in vitro studies
Studies where results were not reported with the PSC reporting system
Sampling other than FNA (e.g., brushings)
Studies reporting cytology sampling of the biliary tract
Studies including less than 30 cases with follow-up
Studies where follow-up was not reported
Inability to extract data
Potential data overlap with already included studies
Follow-up with only a single diagnosis (e.g., pancreatic adenocarcinoma)
Reviews, editorials, conference abstracts, and case reports

**Table 2:** Main characteristics of the studies included in the systematic review.

First Author, Year (Reference)	Country	Lesion Types	Initial Dx or Reclassification	Total No of Patients (F/M)	Mean Age	Total No of FNA Cases	Cases with Follow-Up	Follow-Up Type
Gilani, 2020 [12]	USA	Cystic	Reclassification	120 (72/48)	62	120	120	Histology
Hoda, 2019 [13]	USA	Solid and Cystic	Initial	322 (154/168)	66.1	334	334	Histology and Clinical
Sung, 2019 [14]	USA	Solid and Cystic	Initial	856 (456/400)	67 *	1029	548	Histology and Clinical
Wright, 2018 [15]	UK	Solid and Cystic	Initial	111 (59/52)	63	120	112	Histology and Clinical
Trisolini, 2017 [16]	Italy	Solid and Cystic	Initial	107 (56/51)	67	107	107	Histology and Clinical
Chen, 2017 [17]	China	Solid and Cystic	Reclassification	294 (111/183)	55 *	294	294	Histology and Clinical
Smith, 2016 [18]	USA	Cystic	Reclassification	127 (44/37 in IPMNs; 45/1 in MCNs)	IPMNs: 67 MCNs: 54	127	127	Histology
Layfield, 2014 [19]	USA	Solid and Cystic	NR	317 (NR)	NR	317	317	Histology and Clinical

Abbreviations: IPMNs, intraductal papillary mucinous neoplasms; MCNs, mucinous cystic neoplasms. * These studies reported the median, rather than the mean age.

**Table 3:** Risk of bias of the studies included in the meta-analysis, according to the Quality Assessment of Diagnostic Accuracy Studies 2 (QUADAS-2).

First Author, Year (Reference)	Patient Selection	Index Test	Reference Standard	Flow and Timing
Gilani, 2020 [12]	H	U	U	L
Hoda, 2019 [13]	L	L	U	H
Sung, 2019 [14]	L	L	U	H
Wright, 2018 [15]	L	L	U	H
Trisolini, 2017 [16]	L	L	U	H
Chen, 2017 [17]	L	L	U	H
Smith, 2016 [18]	H	L	U	L
Layfield, 2014 [19]	L	L	U	H

**Table 4:** Risk of malignancy associated with each of the Papanicolaou System categories (I–VI) in the eligible studies of this systematic review. Every column contains the total number cases reported under each category, followed by a parenthesis including the number of cases with a positive outcome (P) and its percentage (highlighted with **Bold**).

First Author, Year (Reference)	I (P, %)	II (P, %)	III (P, %)	IV (P, %)	IVΒ (P, %)	IVO (P, %)	IVO with HGA (P, %)	V (P, %)	VI (P, %)
Gilani, 2020 [12]	6 (2, **33%**)	18 (2, **11%**)	7 (2, **29%**)	68 (14, **21%**)		68 (14, **21%**)	3 (3, **100%**)	5 (5, **100%**)	16 (16, **100%**)
Hoda, 2019 [13]	39 (3, **8%**)	100 (1, **1%**)	25 (7, **28%**)	70 (20, **29%**)	4 (0, **0%**)	66 (20, **30%**)	20 (18, **90%**)	6 (6, **100%**)	94 (94, **100%**)
Sung, 2019 [14]	44 (11, **25%**)	23 (4, **17%**)	86 (36, **42%**)	117 (36, **31%**)	12 (0, **0%**)	105 (36, **34%**)	21 (20, **95%**)	22 (21, **95%**)	256 (255, **100%**)
† Wright, 2018 [15]	9 (2, **22%**)	36 (3, **8%**)	2 (2, **100%**)	18 (0, **0%**)	3 (0, **0%**)	15 (0, **0%**)		4 (4, **100%**)	43 (43, **100%**)
Trisolini, 2017 [16]	18 (10, **56%**)	10 (4, **40%**)	14 (14, **100%**)					15 (14, **93%**)	50 (50, 100%)
Chen, 2017 [17]	21 (12, **57%**)	83 (15, **18%**)	13 (9, **69%**)	20 (4, **20%**)		20 (4, **20%**)		32 (28, **88%**)	125 (125, **100%**)
Smith, 2016 [18]	23 (4, **17%**)	7 (0, **0%**)		89 (17, **19%**)		89 (17, **19%**)	11 (7, **64%**)	5 (4, **80%**)	3 (3, **100%**)
Layfield, 2014 [19]	14 (3, **21%**)	103 (13, **13%**)	23 (17, **74%**)	14 (2, **14%**)				22 (18, **82%**)	141 (137, **97%**)

Abbreviations: I, nondiagnostic; II, negative; III, atypical; IVB, neoplastic—benign; IVO, neoplastic—other; V, suspicious for malignancy; IV, malignant; VI, HGA, high-grade atypia. † In this study, ROM of each category was recalculated using the raw data provided by the authors in the manuscript; histology showing a mucinous cystic neoplasm of any grade, a neoplasm with malignant potential (e.g., neuroendocrine neoplasm of any grade), or a carcinoma was considered a positive outcome. The authors also used the same positive outcome to calculate sensitivity and specificity.

**Table 5:** Sensitivity and specificity of pancreatic fine-needle aspiration cytology reported with the Papanicolaou System, as reported in the eligible studies of this systematic review.

First Author, Year (Reference)	Cytology Categories Considered Positive	Additional Histology Classified as Positive (Besides “Malignancy”)	Sensitivity (%)	Specificity (%)
† Gilani, 2020 [12]	IVO with HGA, V, VI	HGD	61.54	100
	V, VI		53.85	100
	VI		41.03	100
Hoda, 2019 [13]	IVO with HGA, V, VI	HGD, PanNET	92.2	98.8
	V, VI		78.1	100
	VI		66.2	100
‡ Sung, 2019 [14]	IVO with HGA, V, VI	HGD	84.09	98.03
	IVO, V, VI		85.7	61.4
	V, VI		75.8	98.9
	VI		70.1	99.5
Wright, 2018 [15]	IVO, V, VI	PanNET, IPMN, MCN	95.4	100
Trisolini, 2017 [16]	V, VI	PanNET, IPMN	78	85.7
	VI		61	100
Chen, 2017 [17]	V, VI	§ NE	79.27	96.04
	VI		64.77	100
† Smith, 2016 [18]	IVO with HGA, V, VI	HGD	58.33	93.75
	V, VI		29.17	98.75
	VI		12.50	100
Layfield, 2014 [19]	V, VI	§ NE	82.89	93.10
	VI		73.26	96.55

Abbreviations: I, nondiagnostic; II, negative; III, atypical; IVB, neoplastic—benign; IVO, neoplastic—other; V, suspicious for malignancy; IV, malignant; HGA, high-grade atypia; HGD, high-grade dysplasia; PDAC, pancreatic adenocarcinoma; PanNEC, pancreatic neuroendocrine carcinoma; PanNET, pancreatic neuroendocrine tumor; SPN, solid pseudopapillary neoplasm; IPMN, intraductal pancreatic mucinous neoplasm; MCN, mucinous cystic neoplasm; NE, nothing else. † In the Gilani et al. and Smith et al. studies, sensitivity and specificity of all scenarios were calculated using the raw data provided by the authors of the manuscript. In the calculations we added, we did not include the results of the nondiagnostic category. ‡ In the Sung et al. study, sensitivity and specificity in the first scenario (cytology categories considered positive: IVO with HGA, V, VI) were calculated using the raw data provided by the authors of the manuscript. In the calculations we added, we did not include the results of the nondiagnostic category. § In the Chen et al. study, nothing else (no other diagnoses rather than the ones written in the column title) was mentioned as a positive outcome. In the Layfield et al. study, histologic or clinical evidence of malignancy were used as a positive outcome. “Malignancy” included: PDAC, PanNEC, SPN with high-grade malignant transformation, IPMN or MCN with invasion, acinar cell carcinoma, pancreatoblastoma, lymphoma, and metastases.

## Data Availability

Data are contained within the article.
